# Supplementary material for: Piezo1 channels sense whole body physical activity to reset cardiovascular homeostasis and enhance performance
Source: Nat Commun. 2017 Aug 24;8:350. doi: 10.1038/s41467-017-00429-3 (PMC5571199; doi:10.1038/s41467-017-00429-3)
Supplement: Supplementary file 1 — Supplementary Information [file 41467_2017_429_MOESM1_ESM.pdf]

Description of Supplementary Files

File Name: Supplementary Information
Description: Supplementary Figures and Supplementary Table.

File Name: Peer Review File

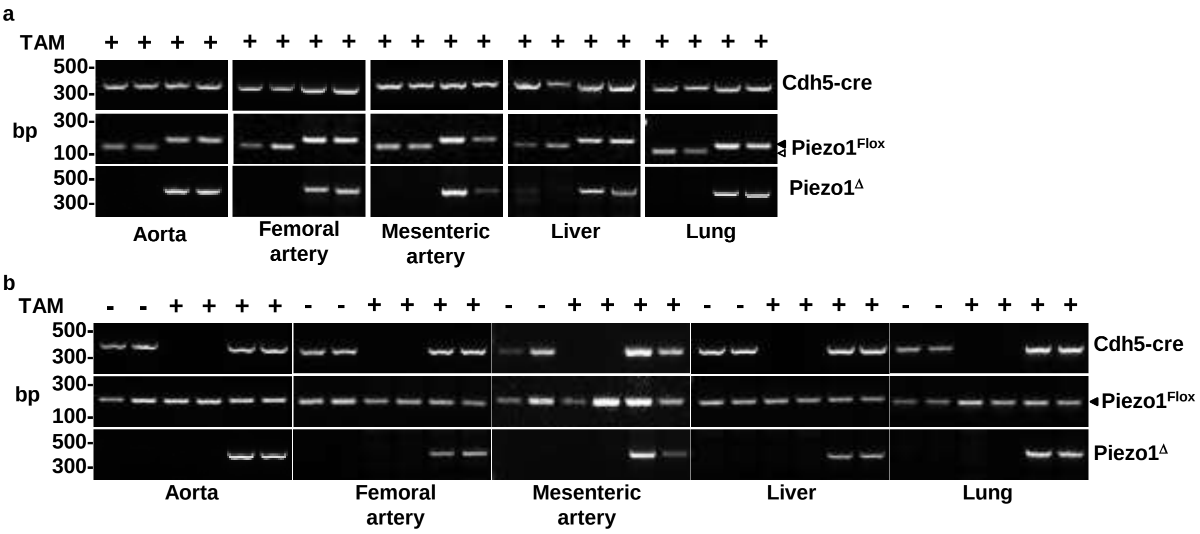

Supplementary Figure 1| Tamoxifen-dependent disruption of *Piezo1* in adult mice. Gel electrophoresis of end-point PCR reactions. Template was DNA from whole tissue (aorta, femoral artery, mesenteric artery, liver and lung). Size of PCR products in base pairs (bp) is indicated on the left. Presence of the cre recombinase transgene is analysed in the top row (Cdh5-cre, 408 base pairs); presence of LoxP sequence in *Piezo1* is analysed in the middle row (*Piezo1*^{Flox}, 155 base pairs when LoxP is absent (white arrowhead); 189 base pairs when LoxP is present (black arrowhead); deletion in *Piezo1* gene is analysed in the bottom row (Piezo1$^{\Delta}$, 379 base pairs). Each column corresponds to one animal, absence (-) or presence (+) of tamoxifen (TAM) injection is indicated above each gel. Each condition is shown in duplicate. (**a**) All mice received TAM and had the Cdh5-cre transgene. The Piezo1$^{\Delta}$ PCR product was not detected when LoxP sequence was absent in *Piezo1* (two left columns) but was detected when LoxP sequence was present in *Piezo1* (two right columns). (**b**) All mice had LoxP sequence in the *Piezo1*. As in (**a**), the Piezo1$^{\Delta}$ PCR product was detected when the mice received TAM injection and had the Cdh5-cre transgene (two right columns) but was not detected when the mice did not have either TAM injection (two left columns) or the Cdh5-cre transgene (two middle columns).

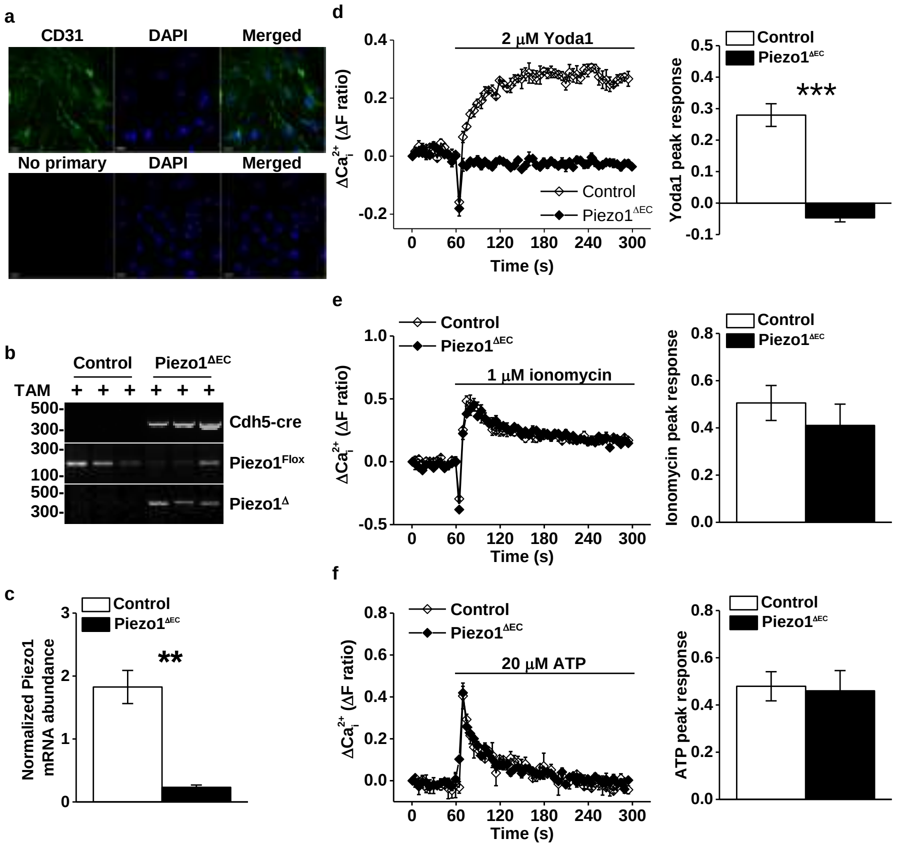

Supplementary Figure 2| Depletion of Piezo1 in endothelial cells of Piezo1$^{\Delta EC}$ mice. Liver endothelial cells (LECs) where isolated from Control and Piezo1$^{\Delta EC}$ mice and cultured without passage. (**a**) Top row: immunofluorescence images of LEC stained with anti-CD31 antibody (green) and DAPI to label nuclei (blue). Bottom row: anti-CD31 antibody was omitted. Scale bars 20 μm. (**b**) Gel electrophoresis of PCR reactions. Template was DNA from LEC isolated from three control mice (three left columns) and three Piezo1$^{\Delta EC}$ mice (three right columns). Presence of the cre recombinase transgene is analysed in the top row (Cdh5-cre, 408 base pairs); presence of LoxP sequence in *Piezo1* is analysed in the middle row (Piezo1^{Flox}, 189 base pairs); deletion in *Piezo1* is analysed in the bottom row (Piezo1$^{\Delta}$, 379 base pairs). The Piezo1$^{\Delta}$ PCR product was detected in DNA from Piezo1$^{\Delta EC}$ mice but not control mice. (**c**) Quantitative RT-PCR analysis of Piezo1 mRNA abundance in LECs from control (n=5) and Piezo1$^{\Delta EC}$ (n=4) mice. (**d**) On the left, representative traces of change (Δ) in intracellular Ca^{2+} in control and Piezo1$^{\Delta EC}$ LECs on application of 2 μM Yoda1. On the right, mean data for the Yoda1 response at 200 s in control (n=6/N=19) and Piezo1$^{\Delta EC}$ (n=6/N=20) LECs. (**e**) On the left, representative traces of change (Δ) in intracellular Ca^{2+} in control and Piezo1$^{\Delta EC}$ LECs on application of 1 μM ionomycin. On the right, mean data for the ionomycin response at 80 s in control (n=6/N=12) and Piezo1$^{\Delta EC}$ (n=4/N=10) LECs. (**f**) On the left, representative traces of change (Δ) in intracellular Ca^{2+} in control and Piezo1$^{\Delta EC}$ LECs on application of 20 μM ATP. On the right, mean data for the ionomycin response at 70 s in control (n=6/N=12) and Piezo1$^{\Delta EC}$ (n=4/N=10) LECs.

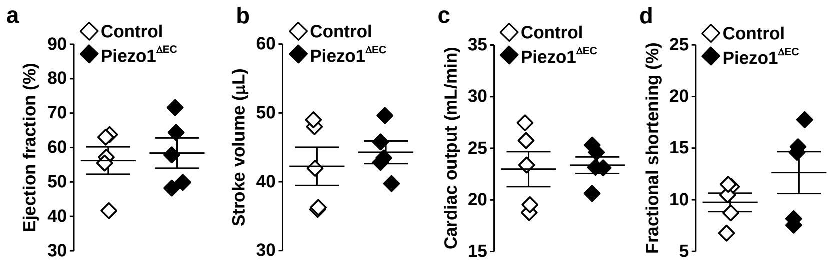

Supplementary Figure 3| Mice with disrupted endothelial *Piezo1* are superficially normal. (**a-d**) Additional data for the ultrasound study of the heart of control (n=5) and Piezo1$^{\Delta EC}$ (n=5) mice under anaesthesia.

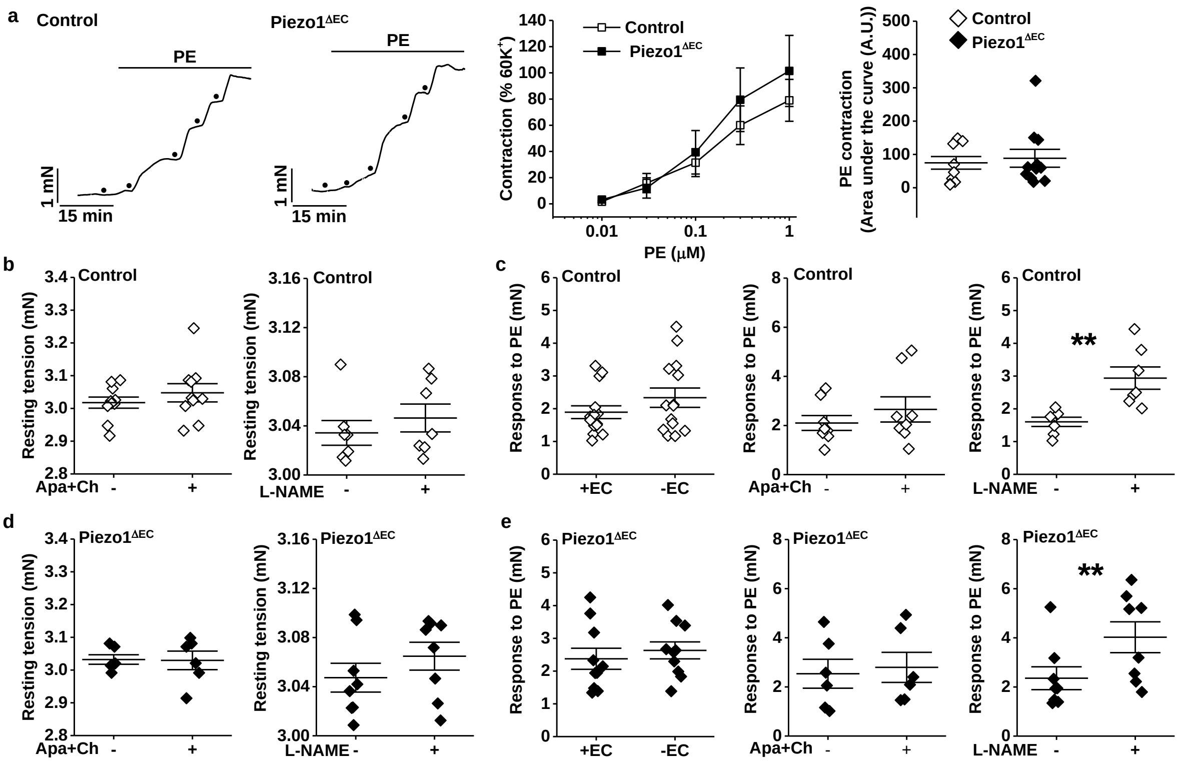

Supplementary Figure 4| Phenylephrine-evoked and resting tension in mesenteric artery. (**a**) Example isometric tension recordings from control and Piezo1$^{\Delta EC}$ mouse second-order endothelium-intact mesenteric arteries. Upward deflection is increasing tension. Phenylephrine (PE) was applied at increasing concentrations as indicated by the dots (0.01, 0.03, 0.1, 0.3 and 1 μM). To the right are mean data for this type of experiment and area under the curve analysis for the same data (n=10 control and n=11 Piezo1$^{\Delta EC}$ mice). (**b**, **c**) Further data for control genotype mice. (**b**) Resting tension in the absence or presence of apamin (Apa, 0.5 μM) plus charybdotoxin (Ch, 0.1 μM) (n=10) or absence or presence of 100 μM nitric oxide synthase inhibitor L-NAME (n=7). (**c**) Contraction evoked by 1 μM phenylephrine (PE) in the presence (+EC) or absence (-EC) of endothelium (n=14), absence or presence of apamin (Apa, 0.5 μM) plus charybdotoxin (Ch, 0.1 μM) (n=8), or absence or presence of 100 μM L-NAME (n=7). (**d**, **e**) Further data for Piezo1$^{\Delta EC}$ genotype mice. (**d**) Resting tension in the absence or presence of apamin (Apa, 0.5 μM) plus charybdotoxin (Ch, 0.1 μM) (n=6) or absence or presence of 100 μM nitric oxide synthase inhibitor L-NAME (n=8). (**e**) Contraction evoked by 1 μM phenylephrine (PE) in the presence (+EC) or absence (-EC) of endothelium (n=10), absence or presence of apamin (Apa, 0.5 μM) plus charybdotoxin (Ch, 0.1 μM) (n=6), or absence or presence of 100 μM L-NAME (n=8).

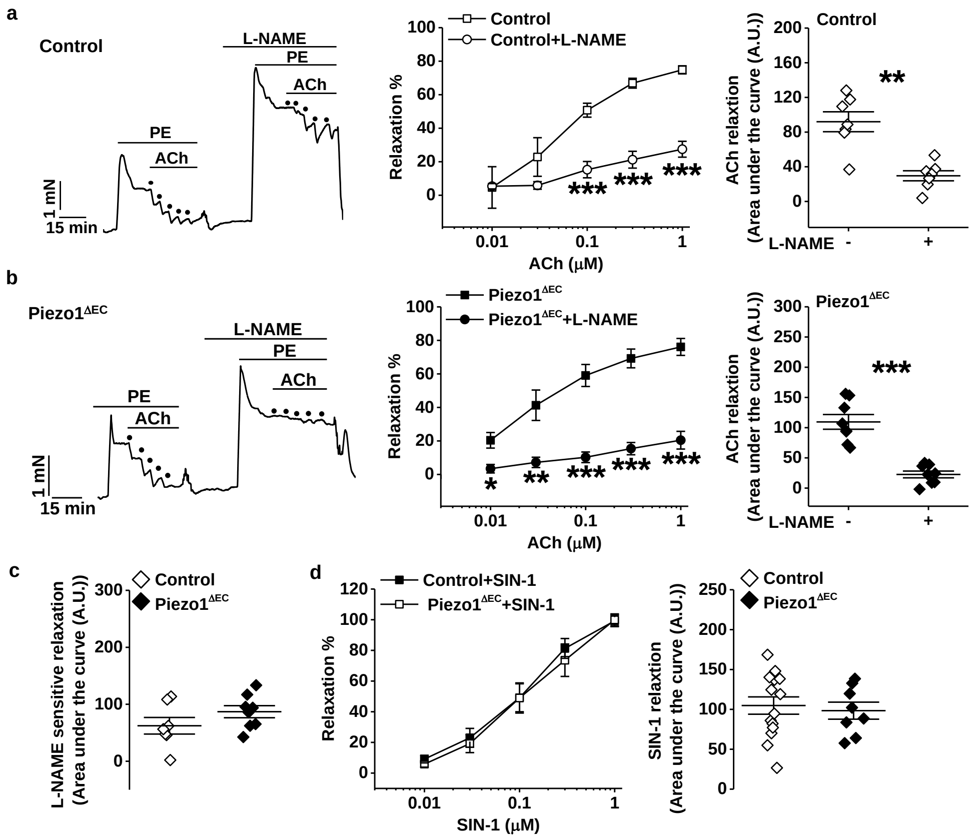

Supplementary Figure 5| Nitric oxide-dependent tension in mesenteric artery. All arteries were endothelium intact. (**a**) Example isometric tension recording from control genotype mouse second-order mesenteric artery before and after addition of 100 μM nitric oxide synthase inhibitor L-NAME. Upward deflection is increasing tension. Phenylephrine (PE, 0.3 μM). Acetylcholine (ACh) was applied at increasing concentrations as indicated by the dots (0.01, 0.03, 0.1, 0.3 and 1 μM). To the right are mean data for this type of experiment and area under the curve analysis for the same data (n=7). (**b**) As for (**a**) but Piezo1$^{\Delta EC}$ genotype mice (n=8). (**c**) Direct comparison of the data from (**a**) and (**b**). (**d**) Concentration-response data for the nitric oxide donor amino-3-morpholinyl-1,2,3-oxadiazolium (SIN-1) for control (n=10) and Piezo1$^{\Delta EC}$ (n=8) genotypes. To the right is comparison of the same data by area under the curve analysis.

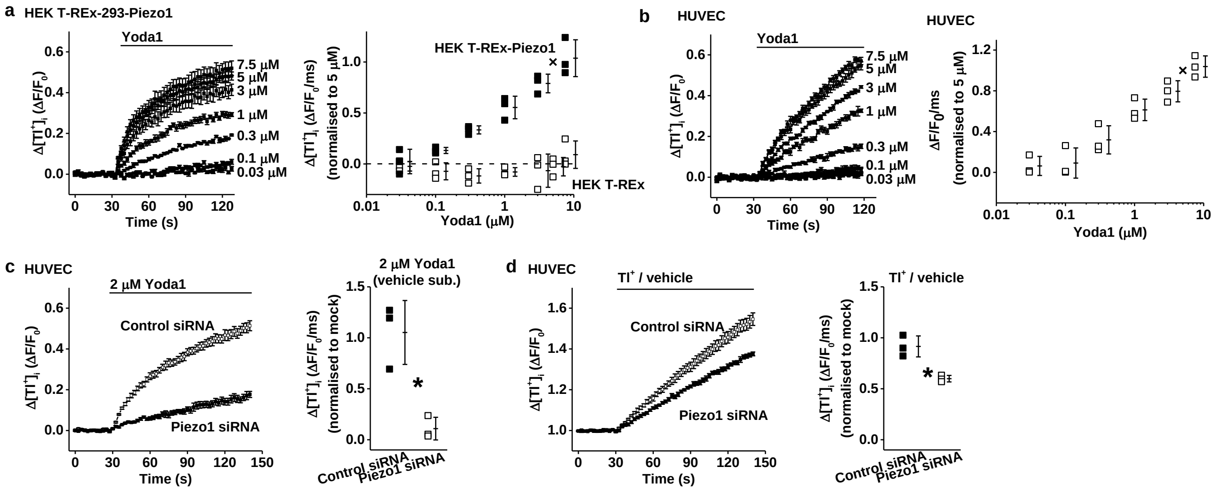

Supplementary Figure 6| Constitutive monovalent cation entry through Piezo1 channels. Unidirectional thallium ion (Tl^+) flux was detected with a Flux-OR^{TM} assay. (**a**) On the left, example traces of Tl^+ influx in modified HEK T-REx-293 cells induced to express exogenous Piezo1 and showing responses to increasing concentrations of Yoda1 (N=4 wells each, mean±s.e.m.). On the right, mean concentration-response data for Yoda1-induced Tl^+ influx in HEK T-REx-293 cells (non-induced controls) and HEK T-REx-293-Piezo1 cells (induced) (n=3 each). Data are normalised to the signal in response to 5 μM Yoda1 in HEK T-Rex-293-Piezo1 cells as indicated by the black cross. Individual data points and the mean±s.d. are shown. (**b**) On the left, example traces of Tl^+ influx in human umbilical vein endothelial cells (HUVECs) in response to increasing concentrations of Yoda1 (N=4 wells each, mean±s.e.m.). On the right, mean concentration-response data for Yoda1-induced Tl^+ influx in HUVECs (n=3 each). Data are normalised to the signal in response to 5 μM Yoda1 as indicated by the black cross. Individual data points and the mean±s.d. are shown. (**c**) On the left, example traces of Tl^+ influx in response to 2 μM Yoda1 in HUVECs transfected with control siRNA or Piezo1 siRNA (N=4 wells each, mean±s.e.m.). On the right, mean data for experiments of the same type (n=3). Individual data points and the mean±s.d. are shown. (**d**) On the left, example traces for constitutive Tl^+ influx in HUVECs transfected with control siRNA or Piezo1 siRNA (N=4 wells each, mean±s.e.m.). On the right, mean data for experiments of the same type (n=3). Individual data points and the mean±s.d. are shown.

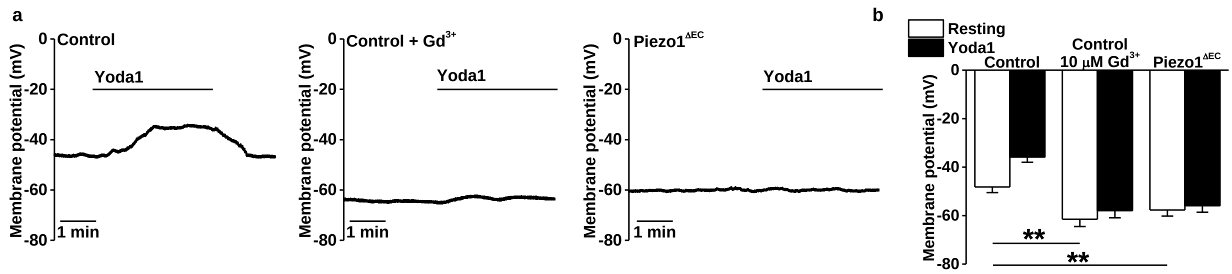

Supplementary Figure 7| Small-molecule activation of Piezo1 channels mimics fluid flow to cause endothelial depolarization. Membrane potential measurements from freshly-isolated endothelium of second-order mesenteric arteries. (**a**) Example traces from the control and Piezo1$^{\Delta EC}$ genotypes in the absence of Gd^{3+} and the control genotype in the presence of 10 μM Gd^{3+}. Endothelium was exposed to 10 μM Yoda1. No flow was applied. (**b**) Bar chart showing mean±s.e.mean values for experiments of the same type as exemplified in (**a**) (n=16 control, n=12 control+Gd^{3+}, n=16 Piezo1$^{\Delta EC}$). Control genotype: no Yoda1 -48.3±2.4 mV *vs* +Yoda1 -35.8±2.2 mV (***). Control genotype in Gd^{3+}: no Yoda1 -61.5±3.1 mV *vs* +Yoda1-57.9±3.0 mV (***). Piezo1$^{\Delta EC}$ genotype: no Yoda1 -56.6±2.6 mV *vs* +Yoda1 -54.7±2.8 mV (**).

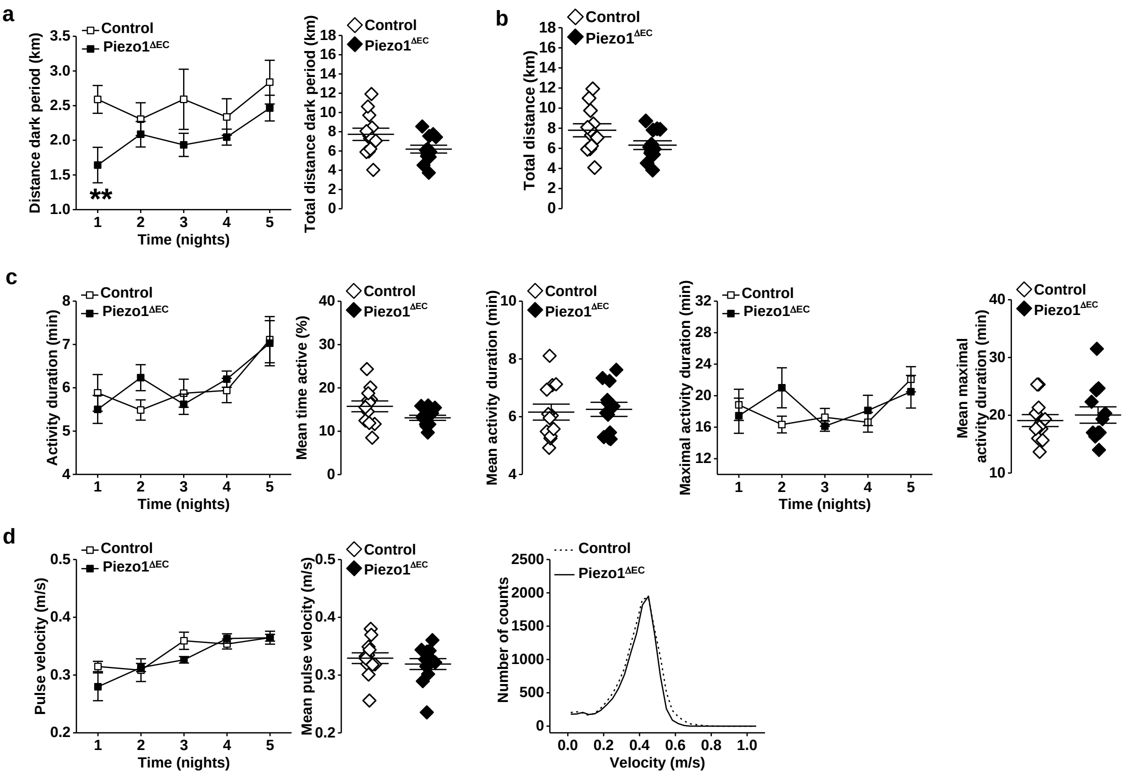

Supplementary Figure 8| Additional whole body activity data. The data show additional analysis of voluntary running wheel usage in control and Piezo1$^{\Delta EC}$ genotypes for the same mice analysed in Fig. 7. Only data for night 1 were compared by *t*-test. (**a**) Distance run on the wheel during the dark period per night and over the total period of analysis. (**b**) Total distance run during dark and light periods. (**c**) Activity duration during the dark period. A mouse was considered to be active when there were ≥2 revolutions of the running wheel during each 1 min recording period. (**d**) Pulse velocity during the dark period. A pulse represents one quarter revolution of the running wheel.

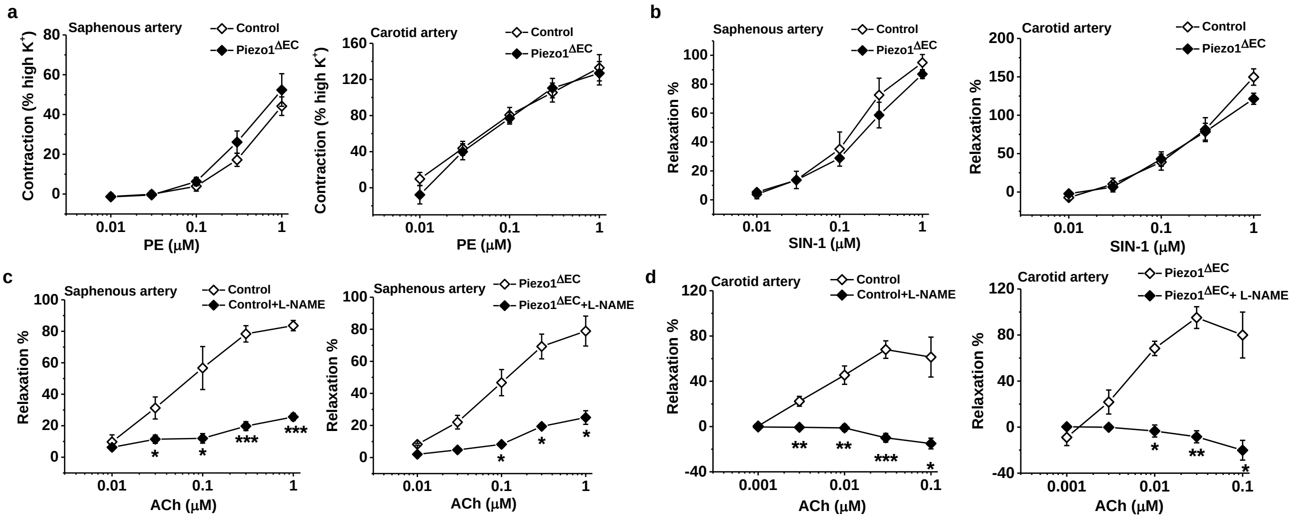

Supplementary Figure 9| Additional isometric tension data for saphenous and carotid arteries. (**a**) Mean data for the contraction response to phenylephrine (PE) as a percentage of the response to 60 mM K^+ and comparing the control and Piezo1$^{\Delta EC}$ genotypes in saphenous artery (n=13 control, n=9 Piezo1$^{\Delta EC}$) and carotid artery (n=15 control, n=11 Piezo1$^{\Delta EC}$). (**b**) Mean data for the relaxation response to the nitric oxide donor amino-3-morpholinyl-1,2,3-oxadiazolium (SIN-1) in arteries pre-contracted with phenylephrine (PE) and comparing the control and Piezo1$^{\Delta EC}$ genotypes in saphenous artery (n=8 control, n=5 Piezo1$^{\Delta EC}$) and carotid artery (n=8 control, n=5 Piezo1$^{\Delta EC}$). (**c**) Mean data for the relaxation response to acetylcholine (ACh) in saphenous arteries pre-contracted with phenylephrine (PE), comparing with and without 100 μM N(ω)-nitro-L-arginine methyl ester (L-NAME) and the control and Piezo1$^{\Delta EC}$ genotypes (n=5 control, n=3 Piezo1$^{\Delta EC}$). (**d**) Mean data for the relaxation response to acetylcholine (ACh) in carotid arteries pre-contracted with phenylephrine (PE), comparing with and without 100 μM N(ω)-nitro-L-arginine methyl ester (L-NAME) and the control and Piezo1$^{\Delta EC}$ genotypes (n=5 control, n=3 Piezo1$^{\Delta EC}$).

Gene	Analysis	Forward primer sequence (5' to 3')	Reverse primer sequence (5' to 3')	Product size (base pairs)
Cdh5-cre	Genomic DNA	GCATTACCGGTCGATGCAA	AGTGAACGAACCTGGTCGA	408
Piezo1^{flox}	Genomic DNA	GGAGGGTTGCTTGTTGGATA	ACTCATCTGGGTGAGGTTGC	155, 189
Piezo1$^{\Delta}$	Genomic DNA	ACCACCTGAGAAGTTGTCCC	ACTCATCTGGGTGAGGTTGC	379
18s	cDNA	GATGCTCTTAGCTGAGTGT	GCTCTGGTCCGTCTTG	233
Piezo1	cDNA	CACAAAGTACCGGGCG	AAAGTAAATGCACTTGACG	370

Supplementary Table 1| PCR primer sequences.
